# Faces on Her and His Mind: Female and Likable

**DOI:** 10.1371/journal.pone.0157636

**Published:** 2016-06-28

**Authors:** Marina A. Pavlova, Annika Mayer, Franziska Hösl, Alexander N. Sokolov

**Affiliations:** 1 Department of Biomedical Magnetic Resonance, Medical School, Eberhard Karls University of Tübingen, Tübingen, Germany; 2 Department of Women’s Health, Women’s Health Research Institute, University Hospital, Eberhard Karls University of Tübingen, Tübingen, Germany; University of British Columbia, CANADA

## Abstract

Faces are a valuable source of non-verbal information for daily life social interaction. Mounting evidence points to gender specificity in face perception. Here we search for the factors that can potentially trigger gender differences in tuning to faces. By using a set of Face-n-Food images slightly bordering on the Giuseppe Arcimboldo style, we examine: (i) whether face resemblance is linked to gender specific face impression, and, if so, whether this association is perceiver gender specific; and (ii) whether images most resembling a face are also most likable for female and male perceivers. First, in a spontaneous recognition task, participants were shown a set of Face-n-Food images in a predetermined order from the least to most resembling a face. Then in a two-alternative forced-choice (2AFC) task, participants judged whether each face appeared for them (i) either female or male (Exp. 1); or (ii) either likable or unlikable (Exp. 2). Remarkably, face resemblance is closely connected to gender specific impressions: images more resembling a face elicit also more female-face responses. This link is not perceiver gender specific as it occurs for both females and males. Moreover, face resemblance is positively linked to face likability, but this holds true only for female perceivers. The findings shed light on gender specificity in tuning to faces, and help to clarify abnormalities of the social brain in neurodevelopmental, psychiatric and psychosomatic disorders.

## Introduction

Faces and bodies provide us with a wealth of socially relevant information [1–6]. Females are widely believed to be more proficient in perception and understanding of non-verbal social communication signals. They are faster in discrimination of emotional from neutral point-light body motion and more accurate in recognition of neutral body motion (such as walking or jumping on the spot) [7]. Magnetoencephalography (MEG) reveals sex specific modes in the brain response to neutral point-light body motion even in the absence of behavioral differences [8]. In females, increased functional magnetic resonance imaging (fMRI) activity is found during viewing point-light body motion (playing pat-a-cake and peek-a-boo) over the regions constituting the social brain [9]. Gender differences in both emotional body language reading and facial affect processing appear to be profoundly modulated by the type of portrayed emotion [10–12].

**Fig 1:**
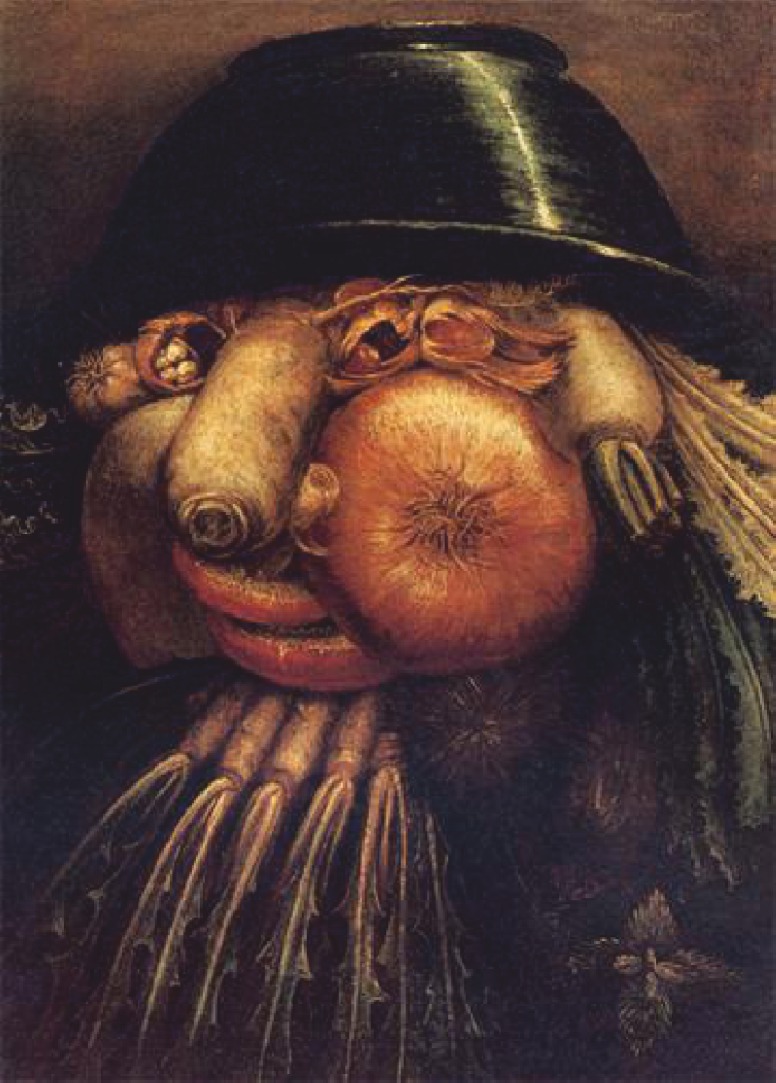
An example of portraits by Giuseppe Arcimboldo. “The Gardner” by Guiseppe Arcimboldo (1526–1593), an Italian painter best known for creating fascinating (often grotesque and allegoric) imaginative portraits composed entirely of fruits, vegetables, plants, tree roots, flowers, and even books and human bodies (http://www.wikiart.org/en/giuseppe-arcimboldo/the-gardner; public domain).

Mounting evidence points to the gender specificity in facial affect recognition and its impairments [3,13]. It remains unclear whether females are better tuned to non-affective faces (as findings are sparse and controversial), though they surpass males in facial identity discrimination, face recognition, and some other aspects of face processing [14–16]. With intention to clarify gender specificity in tuning to faces, a newly created set of Face-n-Food images (composed of food ingredients such as fruits, vegetables, and sausages) had been administered to healthy female and male perceivers [17]. The Face-n-Food images slightly border on the style of Giuseppe Arcimboldo (1526–1593), an Italian painter best known for creating fascinating imaginative portraits composed entirely of fruits, vegetables, plants, tree roots, flowers, and even books and human bodies (Fig 1). Typically developing individuals not only effortlessly see faces in ambiguous Arcimboldo like icons, but also in such images as clouds or ink blots that contain shapes or elements resembling those of a face. This ability is sometimes called *face pareidolia*: perceivers tend to seeing faces almost everywhere. Tuning to faces in the Arcimboldo paintings emerges early in perceptual development: already infants aged 7–8 months prefer the Arcimboldo portraits over the same images presented upside-down [18]. Patients with prosopagnosia (following right unilateral brain damage) or simultanagnosia are capable of perceiving the Arcimboldo faces [19,20]. Recently pronounced gender specificity in tuning to faces has been reported: females recognize the Arcimboldo like Face-n-Food icons as a face more readily than males [17].

Among factors that can potentially affect face sensitivity is gender of a depicted face. Behavioral and neuroimaging research advocates the existence of an opposite-sex bias in face processing [21]. In the same vein, electrophysiological studies demonstrate that components of the event related potentials (ERP) which are thought to represent neural responses to meaningful or motivationally relevant stimuli are larger to opposite-sex as compared to same-sex faces [22]. Functional MRI brain activity in response to faces in the thalamus and medial orbitofrontal cortex is modulated by sexual preference: it is greater in heterosexual men and homosexual women in response to female faces, and in heterosexual women and homosexual men to male faces [23]. Males are reported to be “wired for her face”, exhibiting attentional bias toward female faces, and female faces elicit stronger fMRI brain response in the face sensitive part of the fusiform gyri [24]. By contrast, females are reported to be more proficient at recognizing and remembering female faces exhibiting the own-gender bias [25,26] that is also reflected in the recent meta-analysis [27]. Yet other studies fail to find the own-gender bias in both females and males [16]. Women are not only better in female face recognition, but also show higher activity in response to female than male faces in fusiform and inferior occipital gyri, whereas men's recognition memory and brain activity are not modulated by face gender [28]. On the other hand, it is reported that both male and female perceivers are more efficient in recognition of female as compared to male faces, and several brain areas, including the hippocampal region, exhibit greater fMRI activity in response to female faces [29]. A distributed brain network (engaging the fusiform gyrus, inferior occipital gyrus, superior temporal sulcus, orbitofrontal cortex, and insula) is devoted to decoding gender specific information in faces: it involves the core system that processes invariant facial features, and the extended system that deals with changeable face cues [30]. Overall, so far no consensus is reached as to the issue of how gender cues affect tuning to faces in female and male perceivers.

Earlier studies report that the brows, eyes, whole jaw, chin, nose, mouth, and skin texture carry substantial information on face gender [31–33]. Some researchers conclude that one can judge face gender by the nose shape only; yet male noses are identified better in frontal and in profile views, whereas female noses are identified better in the three-quarter view [34]. Principal component analysis indicates that several cues may affect encoding gender specificity of faces: face complexion (feminine face looks lighter than the masculine one); presence of smiling expression (across cultures, women smile more often); the masculine face is darker around the mouth region; the masculine face has a longer nose; the female face tends to have thinner eyebrows, bigger eyes and lips [35]. But also other cues and their interplay may affect gender related face impressions: for example, skin redness enhances perceived aggression, dominance and even attractiveness in men's faces [36]. Most of these gender cues are minimized or totally absent in the Face-n-Food images [17]. Yet though the images had been created without any purpose to appear gender specific, they can potentially produce gender related impressions.

Other vital factors that play a role in face processing are face likability and attractiveness. It is known that affective components can modify face processing and recognizability [21]. Meta-analyses point to increased activation in the face sensitive part of the fusiform gyrus for attractive as compared to unattractive faces, and this outcome is highly consistent across studies [37,38]. Notably, face attractiveness may intervene face processing as early as the structural encoding stage [39,40]. Regardless of gender and sexual orientation, perceivers similarly rate the attractiveness of both male and female faces [23]. Caucasian and Japanese perceivers prefer (i.e., rate as more attractive) feminized over average female and male faces [41]. Yet people without internet access in El Salvador prefer more feminine male faces and more masculine female faces than people with internet access [42]. The rewarding value of a female face for heterosexual males is discounted by an angry expression: the addition of an angry expression onto an otherwise attractive face renders it aversive to potential mates, albeit mildly happy expressions on the unattractive faces do very little to improve their attractiveness [43]. Overall, likable faces are more readily recognizable and well-remembered than neutral or unlikable ones [44]. Yet it remains unclear whether this holds true for both female and male perceivers. For instance, mechanisms for uncovering face trustfulness are likely to be gender dependent [45].

The present study was aimed at investigation of whether tuning to faces is related to gender of depicted faces and face likability. We intended to clarify the following issues: (i) whether face resemblance is linked to gender specific impressions, and if so, whether this association is perceiver’s gender specific; and (ii) whether images most resembling a face are also more likable for female and male perceivers.

## Methods

### Participants

On overall, one hundred eighteen adults, mostly students and staff of the University of Tübingen, were enrolled in the study. They were assigned to one of three separate groups: pilot, and two experimental. For a pilot study, twenty eight participants (14 females, 14 males) aged 21–54 years were recruited. The group of Experiment 1 included fifty participants (age range, 19–31 years): 25 females, aged 21±1.24 years, median± 95% confidence interval, and 25 males, aged 22±0.95 years; with no gender difference in age (Mann-Whitney test, *U* = 256.5, n.s.). The group of Experiment 2 consisted of 40 participants (age range, 20–28 years): 20 females aged 21.5±0.97 years, median± 95% confidence interval, and 20 males aged 23±0.85 years, with no gender difference in age (Mann-Whitney test, *U* = 263.5, n.s.). Participants were run individually. All participants had normal or corrected-to-normal vision. None had a history of neurological or psychiatric disorders including autistic spectrum disorders (ASD) and attention deficit hyperactivity disorders (ADHD), and regular drugs intake (medication). None had previous experience with such tasks. The study was conducted in line with the Declaration of Helsinki and was approved by the local Ethics Committee at the University of Tübingen Medical School. Informed written consent was obtained from all participants. Participation was voluntary, and the data were processed anonymously.

### Task and procedure

**Fig 2:**
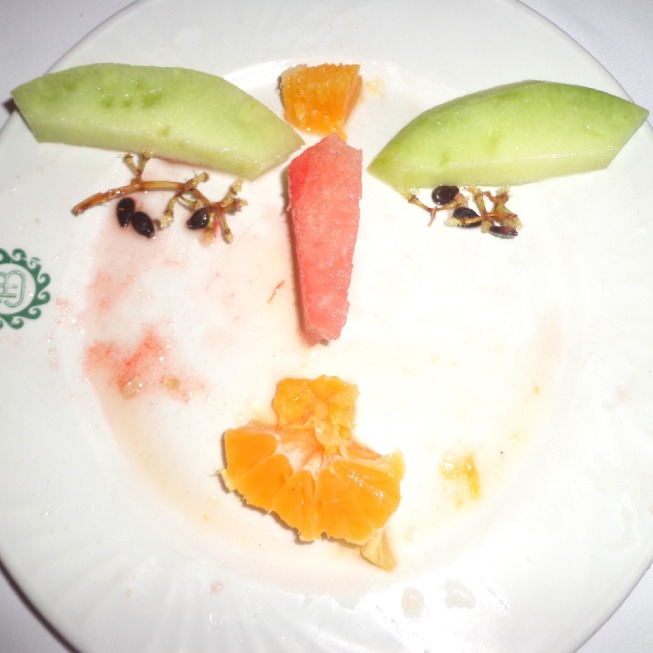
An example of Face-n-Food images. One of the most resembling a face, eliciting most female-face responses, and at the same time, one of the most likable Face-n-Food images.

The Face-n-Food task was administered to participants. For this task, a set of ten images had been created: they were composed of food ingredients (fruits, vegetables, sausages, etc.) and in different degree resembled faces; for details, see [17]. The images slightly border on the Giuseppe Arcimboldo style (Fig 2; for more examples, see [17]). A pilot and two experimental studies had been conducted. The pilot group was presented with a set of images in the predetermined order from the least to most resembling a face (images 1 to 10; the order of stimuli presentation was established earlier [17]). Before testing, they were told that all Face-n-Food images represent faces. On each trial, participants had to perform a two-alternative forced-choice (2AFC) task to determine whether a face appears female or male. In other words, in the pilot study we examined a possible link between gender specific impressions and face resemblance that was determined earlier for the Face-n-Food images by other perceivers [17]. To prove the association between face resemblance and gender specific face impression in the same group of participants, we conducted Experiment 1. In this experiment, participants were shown twelve Face-n-Food images (two additional least recognizable as faces images were added to the set; the order of stimuli was pilot-tested) from the least to most resembling a face. First, participants had to perform a spontaneous recognition task: they were asked to briefly describe what they saw. Then they were told that all images were created to resemble a face. On each trial in a 2AFC task, they were asked to judge whether a face appears to them female or male. In Experiment 2, participants had first to perform a spontaneous recognition task with a set of ten Face-n-Food images. Then they had been told that all images depict faces. On each trial in a 2AFC task, they were asked to judge whether a face appears to them likable or not. Participants had been told that there were no right or wrong responses on these tasks, and they had to rely solely upon their own visual impression. They were run individually. With each participant, the testing procedure lasted for about 20–25 min.

## Results

**Fig 3:**
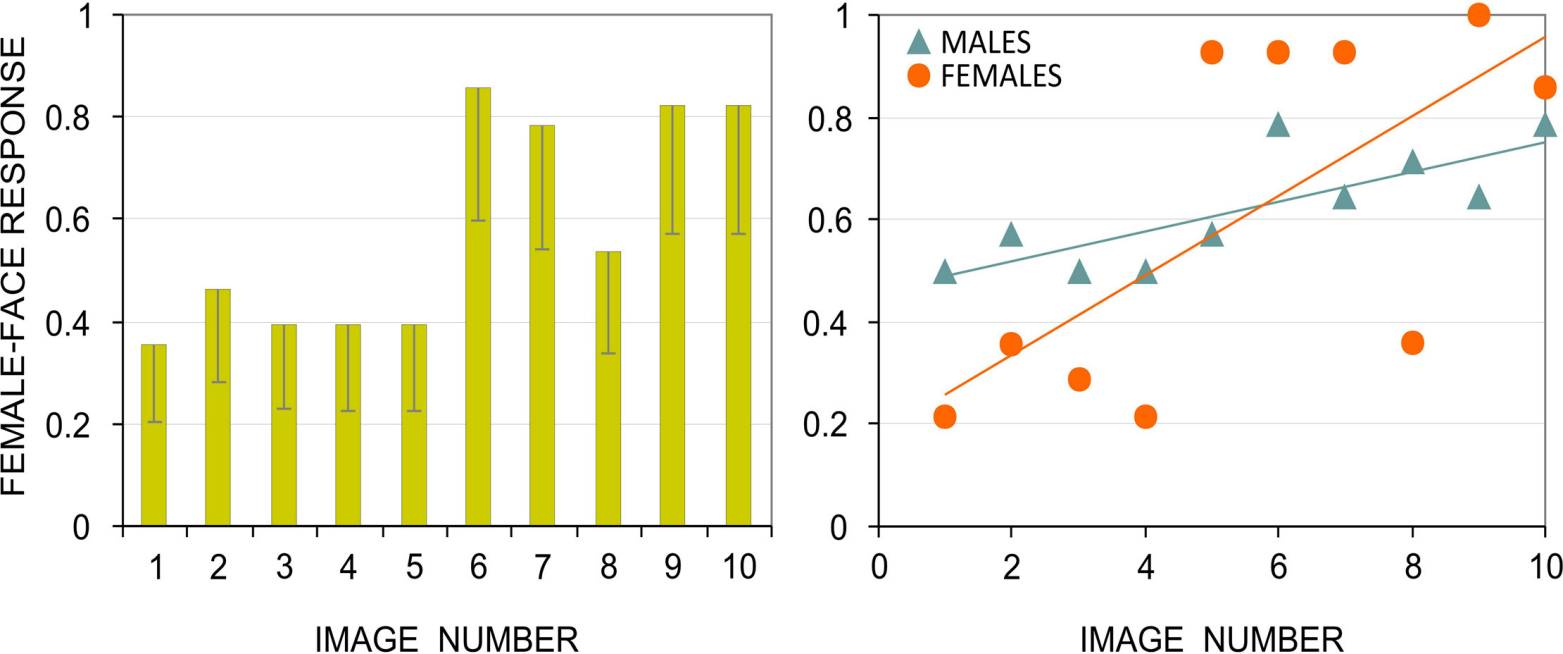
Proportion of female-face responses. Left: Proportion of female-face responses to each Face-n-Food image pooled across female and male perceivers. Vertical bars represent 95% CI (confidence interval). Right: Relationship between face resemblance and female-face impression shown separately for female and male perceivers. The image number reflects its face resemblance (1 –the least recognizable as a face, 10 –the most recognizable); trendlines are shown for illustrative purposes only.

The outcome of the pilot study is represented in Fig 3. As can be seen in this figure (left panel), the Face-n-Food images were not ultimately perceived as gender specific: even when forced to judge the images as a female or male face, participants did not reach any consensus. However, some images did elicit stronger impression of either a female or male face than others. Remarkably, the Face-n-Food images that produced most female-face responses are likely to be more recognizable as a face (Spearman’s rho = 0.776, *p* < 0.008, two-tailed). The question arises whether this link is gender specific or occurs for both female and male perceivers alike. Fig 3 (right panel) represents the outcome separately for females and males. In both females and males, a strong positive link occurred between face resemblance and proportion of female-face responses (Spearman’s rho = 0.654, *p* < 0.04; Spearman’s rho = 0.805, *p* < 0.005; two-tailed, for females and males, respectively). Proportion of female-face responses positively correlated in male and female perceivers (Spearman’s rho = 0.653, *p* < 0.04; two-tailed).

**Fig 4:**
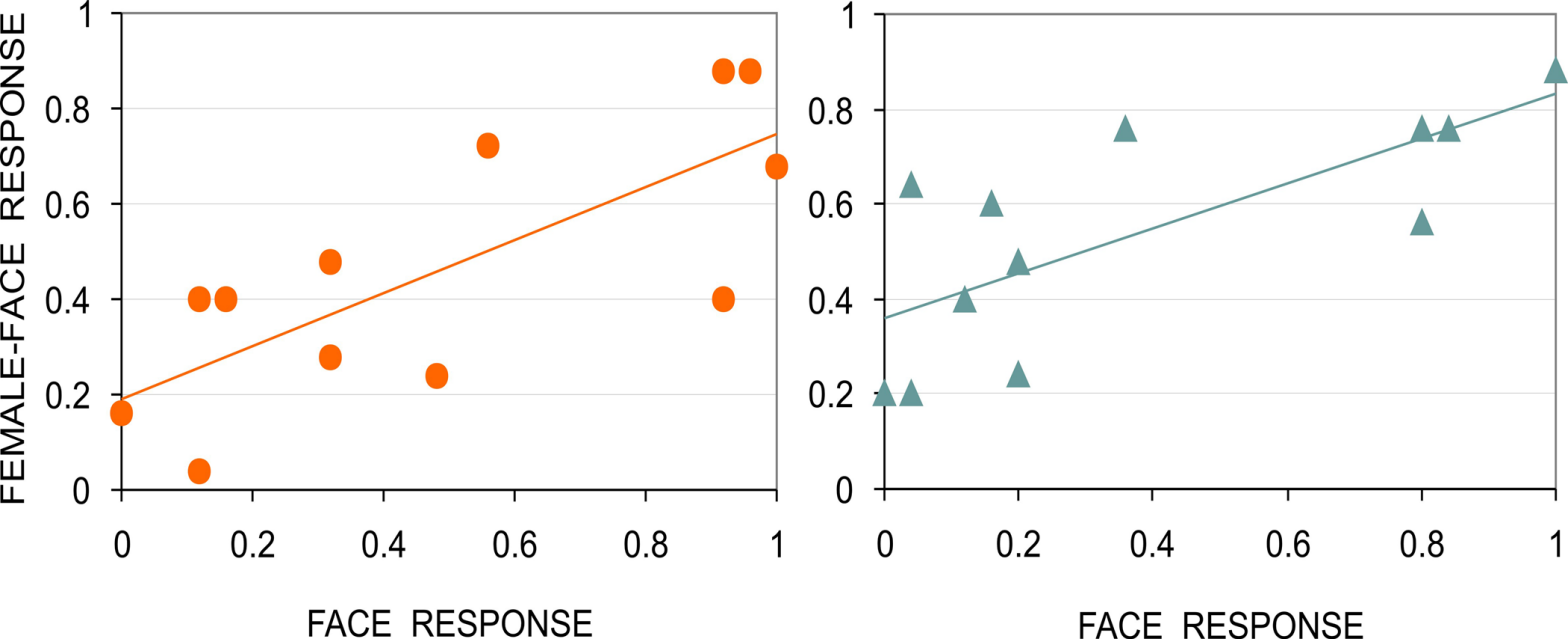
Face resemblance and face gender. Close relationship occurs between gender specific impression (as proportion of female-face responses) and face resemblance (as proportion of face responses) for female, left panel, and male, right panel, participants. In both female and male perceivers, face resemblance positively correlates with female-face impression; trendlines are shown for illustrative purposes only.

In the pilot study, the link occurred between gender specific impressions and face resemblance that was determined earlier for the Face-n-Food images by other perceivers [17]. To prove this association in the same group of participants in Experiment 1, each participant had been asked first to perform a spontaneous recognition task with the Face-n-Food images, and then to judge whether a face appears female or male. As can be seen in Fig 4, for both females and males, the images more recognizable as a face did also elicit more female-face responses (Spearman’s rho = 0.739, *p* < 0.006; Spearman’s rho = 0.761, *p* < 0.004; two- tailed, for females and males, respectively). Proportion of female-face responses in females and males positively correlated with each other (Spearman’s rho = 0.9, *p* < 0.0001; two-tailed).

**Fig 5:**
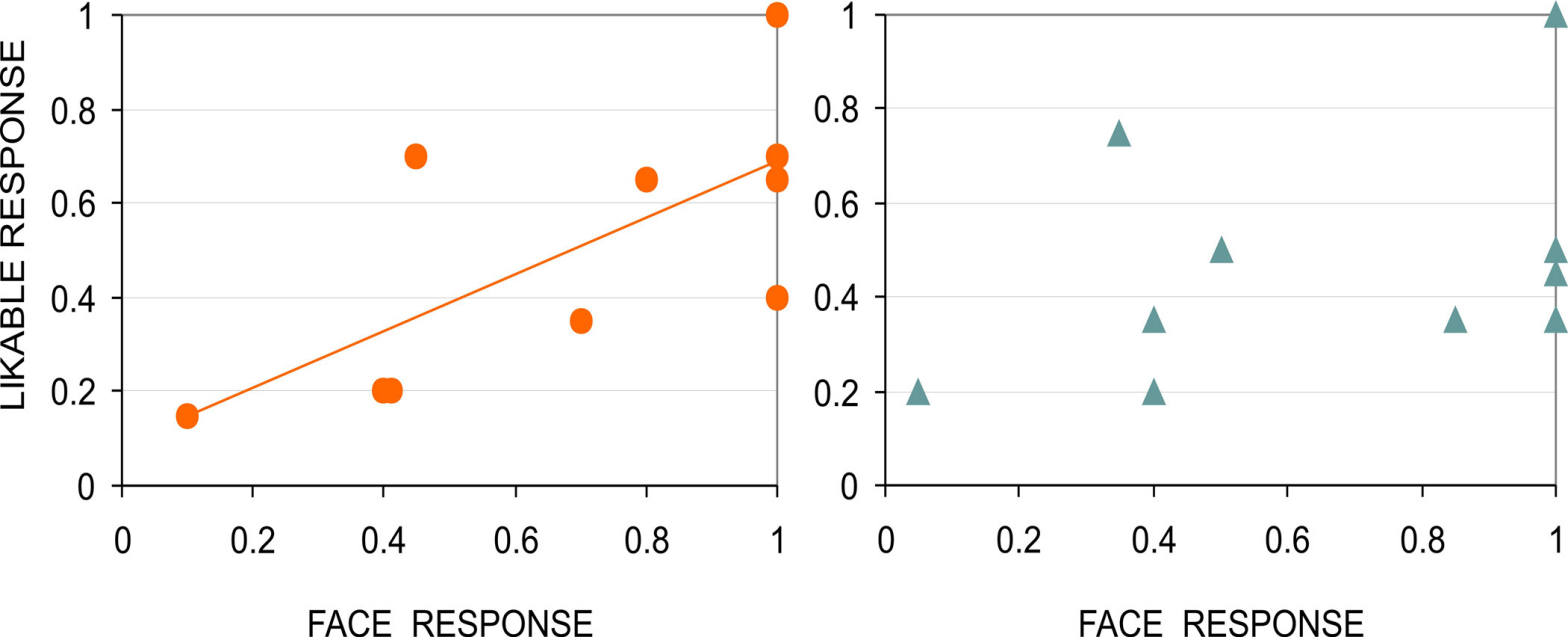
Face resemblance and face likability. Relationship between likability (as proportion of likable-face responses) and face resemblance (as proportion of face responses) for female, left panel, and male, right panel, participants; trendlines are shown for illustrative purposes only.

Finally, the outcome of Experiment 2 (Fig 5) indicated that the Face-n-Food images more recognizable as a face appeared also more likable for females (Spearman’s rho = 0.748, *p* < 0.01, two-tailed). This link, however, was absent in males: face resemblance of the Face-n-Food images was not related to face likability (Spearman’s rho = 0.403, *p* = 0.25, n.s.; two-tailed). Yet face likability judgments in females and males highly correlated with each other (Spearman’s rho = 0.832, *p* < 0.003; two-tailed).

## Discussion

The present study was aimed at investigation of whether tuning to faces is related to gender impression from depicted faces and to face likability in female and male perceivers. By using a recently created set of Face-n-Food images portraying faces as composition of food ingredients (fruits, vegetables, sausages, etc.) in a manner slightly bordering on the Giuseppe Arcimboldo style [17], we searched for the factors that can potentially trigger gender specificity in tuning to faces. The outcome indicates: (i) Face resemblance is closely connected to gender specific face impression: Images more resembling a face elicit also substantially more female-face responses. This link is not gender specific as it occurs in both female and male perceivers. (ii) Face resemblance is positively linked to face likability: Images most resembling a face are also more likable for female, but not for male perceivers. Therefore, tuning to faces is gender specifically related to face likability.

The first upshot is in general agreement with the previous reports that female faces are processed more efficiently [24–29], and this effect is gender independent [29]. At first glance, our findings contradict those indicating that gender-opposed faces are processed easier [21–23], or female faces are processed more efficiently in female perceivers only [24–28]. One possible explanation for this apparent controversy is that different studies address various stages of face encoding. As mentioned earlier [17], methodological issues (the nature of stimuli: real (photographs and movies), depicted or arty faces; different stages of face processing addressed; task demands that may be non-specific to face processing itself) may be of potential value for the outcome of research aimed at uncovering gender specificity in tuning to faces. It appears that tasks tapping affective, motivational or attentional components of face processing more often result in the outcome that gender-opposed faces are processed more efficiently. With ambiguous Face-n-Food images, it is likely that image components resembling salient face cues (e.g., big “eyes”, sunny “smile”, specific “nose” shape, and plumpness) and their interplay that is essential for recognition of an image as a face, are also associated with ladylike face impression. In line with this, when perceivers are in doubt judging face gender on the male-female range of the test morph continua (where salience of face cues is minimized), they express a tendency to judge images as male faces [46]. As the link between face resemblance and ladylike face impression found in the present study occurs in both female and male perceivers, it is unlikely that gender impression alone can account for better face tuning in females reported earlier on the Face-n-Food task [17].

To the best of our knowledge, for a first time we show gender specificity of the link between face likability and face resemblance: association between face responses and face likability is observed in females, but not in male perceivers. Although likable faces are more readily recognizable and well-remembered than neutral or unlikable ones [44], it remained unclear whether this holds true for both females and males. As affective components can modulate cognitive brain networks [21,47], present findings suggest that face likability can at least partly facilitate tuning to faces in females. Future research and, in particular, brain imaging should contribute to further understanding of this issue.

Finally, our data indicate that face likability judgments in female and male participants exhibit a strong positive correlation with each other. This findings is in general agreement with the previous reports showing that regardless of gender, perceivers similarly rate the attractiveness of both male and female faces [24], and prefer feminized faces [43].

As face resemblance increases with both likability and female-face impression in females, one can assume that face likability and gender impression are linked in female, but not in male perceivers. Yet clarification of this issue requires special experimental proof that is beyond the scope of this study.

Among other factors, perceptual (local or global/holistic) style [48,49] can contribute to face tuning in part-whole ambiguous Arcimboldo-like images. One can perceive an image either as a composition of elements (fruits, vegetables, etc.) or as a whole (face), though once seen as a face, Arcimboldo paintings are processed with a strong face-dominating bias. Recent findings indicate that original Arcimboldo hidden-face portraits are judged as being more ambiguous by perceivers with local than global perceptual style, and this effect is absent when viewing Renaissance portraits [50]. Females (but not males) with a local perceptual style judge Arcimboldo artwork as more aesthetically pleasant than do females with global style [50]. One possible account for this effect is that ambiguous paintings are preferred over non-ambiguous ones: in watching abstract artwork, ambiguity is associated with greater pleasure and interest, though ambiguous images are harder to process [51,52]. The other option may be that Arcimboldo portraits are allegoric, often bizarre and grotesque (and sometimes ugly; Fig 1), and therefore can be experienced as unpleasant. In accord with this explanation, the occipito-temporal network engaged in face processing (including the fusiform gyri, parahippocampal gyri, and the inferior temporal gyri) are greater activated when perceivers dislike Arcimboldo artwork, but like Renaissance portraits and non-artwork non-ambiguous and ambiguous face depictions [53].

The present work further contributes towards putting the Face-n-Food task into clinical setting. This task benefits also from using unfamiliar images that is of importance in clinical settings [54]. Most neuropsychiatric, neurodevelopmental and psychosomatic disorders are characterized by impairments in visual social cognition, non-verbal communication, body language reading, and facial assessment of a social counterpart [4,5,55,56]. In turn, most diseases related to impairments in visual social cognition are gender-specific: females and males are differently affected in terms of clinical picture, prevalence, and severity. A wealth of brain imaging and neuropsychological work suggests that various aspects and stages of face processing are deficient in most of these disorders [57]. Face recognition is stronger impaired in autistic males [58] and in survivors of preterm birth [59,60]. Several aspects of face processing are compromised in females with major depressive disorder (MDD) [61], and with social anxiety [62,63]. Pronounced gender differences in face processing along with brain processing of face affect are also reported in eating disorders such as anorexia nervosa and bulimia nervosa [64–66].

In the typical brain, the time-course of the MEG response in the right ventral fusiform face area (FFA), a hub of the social brain for handling face information, during processing of faces and face-like ambiguous images is rather similar: the brain is likely to be hardwired to detect the presence of a face as quickly as possible, rather than to process face-like images later on under influence of top-down mechanisms. Yet the right superior temporal sulcus (STS) differentiates between faces and face-like images [67]. Brain imaging (e.g., fMRI) also indicates that the right FFA is active during perception of noise images containing components resembling a face: images even with the slightest suggestion of a face are interpreted as faces [68].

Only a few brain imaging studies investigated brain response to Arcimboldo portraits, and the outcome, in particular, in respect to topography and hemispheric dominance of activation, appears rather controversial. Arcimboldo paintings compared to Renaissance portraits and non-artistic face representations (photographs) elicit greater fMRI response in the occipito-temporal network dedicated to face processing (including the FFA) as well as in the right inferior frontal gyrus and bilateral superior and inferior parietal lobule [53]. Compared to the same inverted (upside-down) presentations, upright Arcimboldo portraits activate the right FFA and posterior STS [69]. Electroencephalography (EEG) indicates that in the right brain hemisphere, face-sensitive N170 component of the ERP is the same in response to natural faces and Arcimboldo portraits, but over the left hemisphere N170 amplitude is larger for natural faces [70]. Near-infrared spectroscopy (NIRS) in 7–8 month-olds indicates that the left temporal area of the brain is more responsive to the Arcimboldo portraits than to single elements (vegetables) [18].

Much closer look at specific topographic patterns and temporal dynamics of the neural circuitry underpinning facial processing (with hubs in the FFA and STS, which are considered pivots of the social brain) can add essential information on typical and atypical processing of Face-n-Food images. For uncovering face processing in the social brain, one can take an advantage of ultra-high field fMRI providing for high sensitivity and spatial resolution along with concurrent EEG recording to simultaneously obtain precise spatial and temporal information.

## Conclusions

We searched for the factors that can potentially trigger gender specificity in tuning to faces. Remarkably, face resemblance is closely connected to gender specific impression: images most resembling a face elicit also more female-face responses. This link is not perceiver’s gender specific as it occurs for both females and males. Moreover, face resemblance is positively linked to face likability, but this holds true only for female perceivers: in females, images most resembling a face appear also more likable. The findings shed light on gender specificity in tuning to faces, and help to further clarify social brain abnormalities in neurodevelopmental, psychiatric and psychosomatic disorders.
